# Sequential Extractions and Toxicity Potential of Trace Metals Absorbed into Airborne Particles in an Urban Atmosphere of Southwestern Nigeria

**DOI:** 10.1155/2018/6852165

**Published:** 2018-03-01

**Authors:** Emmanuel Gbenga Olumayede, Thompson Faraday Ediagbonya

**Affiliations:** ^1^Department of Industrial Chemistry, Federal University Oye Ekiti, Oye-Ekiti, Nigeria; ^2^Department of Chemical Sciences, Ondo State University of Science and Technology, Okitipupa, Ondo, Nigeria

## Abstract

The paper investigates the hypothesis that biotoxicities of trace metals depend not only on the concentration as expressed by the total amount, but also on their geochemical fractions and bioavailability. Airborne particles were collected using SKC Air Check XR 5000 high volume Sampler at a human breathing height of 1.5–2.0 meters, during the dry season months from November 2014 to March 2015 at different locations in Akure (7°10′N and 5°15′E). The geochemical-based sequential extractions were performed on the particles using a series of increasingly stringent solutions selected to extract metals (Cd, Cu, Cr, Ni, Pb, Zn, and Mn) into four operational geochemical phases—exchangeable, reducible, organic, and residual—and then quantified using an Atomic Absorption Spectrophotometer. The results showed metals concentration of order Pb > Cr > Cd > Zn > Ni > Cu > Mn. However, most metals in the samples exist in nonmobile fractions: exchangeable (6.43–16.2%), reducible (32.58–47.39%), organic (4.73–9.88%), and residual (18.28–27.53%). The pollution indices show ingestion as the leading route of metal exposure, with noncarcinogenic (HQ) and cancer risk (HI) for humans in the area being higher than 1.0 × 10^−4^, indicating a health threat.

## 1. Introduction

Dust is simply the finely divided particulate matter that can be readily lifted by wind from its place of origin and carried along by the turbulent atmospheric currents, finally settling either by gravity under calm condition or by being brought to the surface by precipitation [1]. Worldwide, nonoccupational exposure to dust especially in urban centres has aroused much attention because it is associated with some trace metals and diseases. Dust is generated during human activities such as sweeping, quarrying, and metals fabrication. Once generated and released to the atmosphere, it travels long distances from the emission source. In the course of transportation, the particulate is bound to metals; hence, it creates a reserve pool of metals in urban atmosphere [2]. The presence of these metals in dust particles poses a significant environmental risk to the people.

On breathing air containing metal absorbed into particulate matters, some of the smaller particles reach the wind pipe (trachea) and eventually dissolve in the blood stream. According to [3], the two most serious health problems caused by dust are cancers of the lungs, throat, and nose and other lung conditions called chronic obstructive pulmonary disease (COPD) that includes chronic bronchitis and emphysema. A report in 2007 gave a likely figure between 7 and 8,000 for cancers of the lung and the nose that are due to work exposure to dust [2].

Quite a lot of researchers have investigated elemental compositions of suspended particulate matters in cities worldwide: Monterrey, Mexico [4], Tehran, Iran [5], and Nigerian cities [6–10]. In most of these studies, elevated levels of heavy metals have been observed in atmospheric suspended dust in most cities. Okunola et al. [8] reported the presence of heavy metals such as Cd, Cr, Ni, Pb, Cu, and Zn in atmospheric settling dust in Kano metropolis of Nigeria. Meanwhile, Mafuyai et al. [9] reported that the concentrations of some heavy metals were found to be far above the standard limits prescribed by WHO for respirable dust in Jos, Nigeria.

Meanwhile, it has been hypothesized that the biotoxic effect of metals depends not only on the concentration as expressed by total amount but also on their bioavailability, origin, and properties [11]. To gain a deeper understanding of risk posed by individual metal, it is necessary to determine bioavailability of the metals in different geochemical phases. A lot of investigators [7, 12, 13] have employed sequential extractions to assess metal labiality and to characterize the speciation of metals in solid phases. It has also been suggested that the most easily mobilized metals presented a risk to plants and animal [14]. However, in most cases the most abundant metals are not always the most mobile metals suggesting that other mechanisms are important in the uptake process.

As studies continued to unravel the composition of dust in urban atmosphere, we have devoted the study reported in this contribution to investigating the geochemical-based patterns and toxic potential of some trace metal contents of aerosols particles samples in a fast growing city of Akure (7°10′N and 5°15′E). The city is situated in the rainforest zone of southwest Nigeria, with a population of about 360,268 [15]. It is one of the fastest growing urban settlements in southwestern Nigeria, serving as transitory link to northern and eastern parts of the country, with many industrial plants. The climatic conditions of Nigeria and Akure have been described elsewhere. The objectives of this study include (i) assessing the contamination level of the monitoring sites, (ii) determining the geochemical association of the studied metals along the various phases, (iii) investigating the mobility of metals found in airborne particles collected, and (iv) assessing the carcinogenic and noncarcinogenic toxicity potentials of the metals on the basis of relative content of the fractions. It is envisaged that the data generated will stimulate environmental concerns on the impacts of airborne particulates and translate them into improved respiratory health among populace exposed to dust.

## 2. Materials and Method

### 2.1. Sampling Sites and Sampling Protocols

The sampling sites (different human-impacted environmental settlings) for this study were carefully chosen based on preliminary air quality assessment, using the pollution load index (PLI), and calculated using the method developed by [19]

(1) $$\begin{array}{c} PLI=\sqrt[{n}]{{CF}_{1}+{CF}_{2}+{CF}_{3}+\cdots +{CF}_{n}}, \end{array}$$

where CF is the contamination factor calculated according to [20, 21].

The description, coordinates, and air quality of the sampling sites are shown in Table 1. Airborne particles were collected in selected sampling sites within Akure and in an unpolluted (background) area, at human breathing height of 1.5–2.0 m, twice per month from November 2014 to March 2015. The sampling unit is a portable SKC Air Check XR 5000 high volume Gravimetric Sampler that consists of a gas pump, a Whatman glass fiber filter, Teflon tube, and gas flow rate meter of 1000–5000 mL/min. The unloaded glass fiber filters were dried in desiccators at room temperature. The particulates were collected on the preweighed filter by pumping 2000 mL/min (2 L/min) volume of air through it for eight (8) hours. After sampling, the particles accumulated on the filter paper were removed by tapping on glazing paper and we determined the amount of particles (gm^−3^) on the loaded filters.

### 2.2. Chemical Analysis

The pseudo-total metal concentrations were determined after digestion of one gram (1.0 g) of the collected particles with mixture of 15 ml conc. HCl and 25 ml HNO3 for 2 hrs at 100°C [22]. After cooling, the mixture was filtered and made up to 50 ml volume before extraction. The extraction protocols involved series of increasingly stringent solutions selected to extract metals (Cd, Cu, Cr, Ni, Pb, Zn, and Mn) into four operational geochemical phases: exchangeable (1 M MgCl_2_, pH 7, 1 h), reducible (mixture of NH_2_OH, HCl, and 25% CH_3_COOH, 96°C, 6 h), organic (mixture of HNO_3_, 30% H_2_O_2_, 85°C, 5 h), and residual (mixture of 40% HF, 72% HClO_4_, and conc. HNO_3_ for 4 h). The protocol was adopted from published methods [19, 21]. The metals content in the filtrates were analyzed by Bulk Scientific (model 200A) Atomic Absorption Spectrophotometer (AAS) using air-acetylene flame at optimum wavelength of each metal. Standards used to calibrate the AAS were obtained as commercial BDH stock metal solutions from which working standards were prepared by appropriate dilution.

### 2.3. Quality Control

The quality control of the methods includes the analysis of some samples spiked with known standard and subjected to some process so as to determine the accuracy and precision of the method used. The percentage recoveries were calculated after analysis of the BCR701, lake sediment certified for extractable trace elements, and reference material given as follows:

(2) $$\begin{array}{c} \%\;\text{recovery}=C_{sp}-\frac{C}{C_{a}}, \end{array}$$

where *C*_sp_ is the spike sample concentration, *C* is sample concentration, and *C*_*a*_ is the added concentration.

### 2.4. Data Analysis

The “Analysis Toolpak” available in Microsoft Office Excel 2007 and SPSS version 14 software provides data analysis. Data obtained were also subjected to analysis of variance (ANOVA) to enable us to assess the levels of significant difference.

### 2.5. Determination of Contamination Index

The contamination index (pollution index-*P*_*i*_) of the metals in the sampled particles was obtained by dividing the total concentration obtained by its maximum allowed limit (MAL) [22].

### 2.6. Determination of Mobility of the Metals in Dust Samples

The mobility factor (MF) of metals in sampled particles was calculated using [23]

(3) $$\begin{array}{c} \text{MF}=\frac{\text{Exchangeable}\times 100}{\text{Exchangeable}+\text{reducible}+\text{organic}+\text{residual}}. \end{array}$$

The average mobility factors (MF) are calculated as a percentage of the concentration of metal in the exchangeable fraction and its pseudo-total concentration (sum of metal concentration levels in all the four fractions).

### 2.7. Toxicity Assessment of the Metals

The environmental risk factor (ERF) for metals was determined using

(4) $$\begin{array}{c} ERF=CSQV-\frac{C_{i}}{CSQV} \end{array}$$

(see [24]), where CSQV is the metal concentration in residual fraction and *C*_*i*_ is the metal concentration in the first three fractions.

### 2.8. Human Exposure Risk Assessment

In order to evaluate the potential health impact on the population in the area via inhalation, ingestion, and dermal contact, we calculated the estimated daily intakes (EDIs) of the trace metals in particles, based on the model developed by [25]. The estimated daily exposure doses (*D*) through the three routes were determined using (5)–(7):

Dose contact through ingestion (mg kg^−1^ day^−1^)
  (5) $$\begin{array}{c} D_{Ingestion}=C\times \frac{\left(IRc\times ED\times EF\right)}{BW\times AT}\times 10^{-6} \end{array}$$
(see [26]).
Dose contact through inhalation (mg kg^−1^ day^−1^)
  (6) $$\begin{array}{c} D_{inhalation}=C\times \frac{\left(EF\times {IR}_{inh}\times ED\right)}{PEF\times BW\times AT} \end{array}$$
(see [26]).
Dose contact through dermal contact (mg kg^−1^ day^−1^)
  (7) $$\begin{array}{c} D_{Dermal}=C\times \frac{\left(SA\times SL\times ABS\times EF\times ED\right)}{BW\times AT}\times 10^{-6} \end{array}$$
(see [26]).

Where, (see Table 2).

The potential health risk index for each of the suspected metals was characterized by hazard quotient (HQ) for both cancer risk and noncancer risk.

(8) $$\begin{array}{c} \text{Hazard Quotient }\left(\;HQ\;\right)=\frac{D\times RB}{RfD}; \end{array}$$

see [26].

## 3. Results and Discussion

The result of the preliminary study on degree of dust contamination at the various sampled sites in Akure (Table 1) showed that three categorizes of pollution index were found in the city. However, pollution index (*P*_*i*_) at each site reflects the living pattern of the people or human activities in the area. This observation supports the earlier assertion of Olumayede and Okuo [27] that atmospheric quality in Nigeria urban centres reflects the living pattern of the people.

Firstly, we measured the variation of total metals and the results were presented in Table 3. It is interesting to note that ANOVA test shows that there exists a significant (*p* < 0.05) spatial variation of metal levels in airborne dust among the sites. It can be observed that the pseudo-total concentration of the metals ranked in the order Pb (11.82–70.49) > Cr (9.02–18.57) > Cd (3.05–10.63) > Zn (1.38–11.12) > Ni (0.87–5.55) > Mn (0.60–5.81) > Cu (0.54–3.54). The high concentrations of Pb and Cr are an indication that the particulates originated from anthropogenic emissions and possibly from vehicular emission in the area. Meanwhile, high concentration of Cr could be attributed to weathering of local geological materials. Cr is one of the commonest elements in earth crust and exists in ores such as chromite (FeCr_2_O_4_) and cryolite (FeCrO_4_). The possible anthropogenic sources of Cr in Akure atmosphere include combustion of fuels, metallurgical processing, waste incineration, welding, chrome plating, and spray painting.

To confirm that the total concentration of a metal does not suffice in predicting bioavailability and environmental risk, we hypothesized that metals availability in dust particles should follow a decreasing order from the first step toward the last step in a sequential extraction [28]. We attempted the fractionation and validation of the sequential extraction steps. The results of the certified and found values of the analytical method in the various steps are presented in Table 4. The results also showed good agreement between the found and certified values in most steps. The percentage recovery levels were within 100 ± 5% in all the steps.

**Figure 1:**
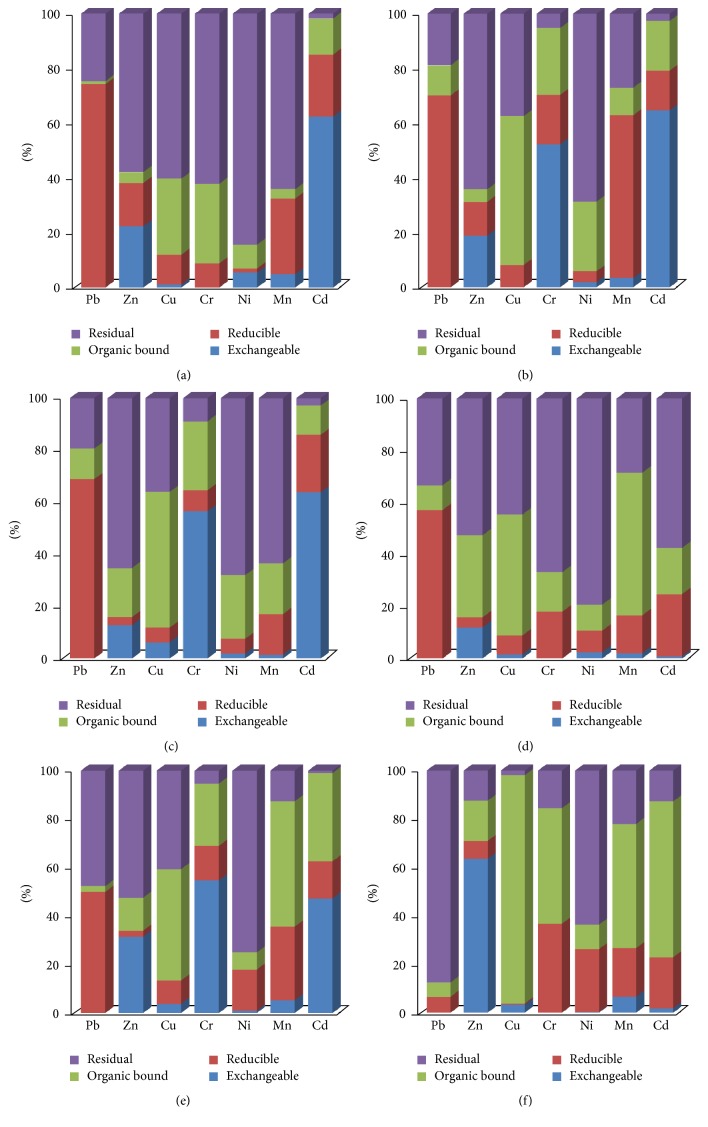
Distribution of metals in different geochemical fractions in dust samples at the sampling sites within Akure Metropolis. (a) AKOB; (b) AKOA; (c) AKEX; (d) AKAL; (e) AKOR; (f) AKBG.

Figure 1 presents the concentrations of metals in different geochemical fractions of the particulates sampled. The results indicated that chemical association of metals varied significantly (*p* > 0.05) among the sites and the association showed no clear order in the entire sampling sites.

One striking feature in the distribution of the metal is that at all sampling sites, Pb has the highest concentration at the reducible fraction with the percentage range between 40 and 60%. The environmental implication of this is that it has high mobility and bioavailability. Further to this is that Pb, Cu, and Cr were not detected in exchangeable fraction in most sites.

The high percentages of Zn and Ni were observed in the residual fraction at traffic impacted sites like AKEX, AKOB, and AKOR. The major sources of these metals include electronic wastes, traffic emission, or wearing of tire. The significance of high amounts of Zn in this fraction agrees with earlier observation of Okunola et al. [8] that the metal is considered to be occluded inside crystalline structure and not readily available for absorption. This observation is also consistent with earlier reports in urban street dust in Nigeria. The existence of Ni in this fraction showed that the metal is strongly bounded to the resistant part of the dust particles hence it is considered stable.

In organic bound fraction, Cu predominated ranging from 54% to 74.8% in all the sites. Metals associated with organic bound fraction are not considered mobile or available and they are associated with high molecular weight stable humic substance. The predominance of Cu in dust samples in organic fraction in this study indicates high organic or sulphide wastes in the studied areas.

The maximum levels of Cd and Cr (32.3–58.9%) were observed at exchangeable fraction at AKOB and AKOR sampling sites. The implication of this is that these metals pose serious environmental risk in such area.

Generally, the range of 6.43–16.2% of the pseudo-total metals is bound to exchangeable fraction, 32.58–47.39% bound to reducible fraction, 4.73–9.88% bound to organic fraction, and 18.28–27.53% bound to residual fraction.

On comparing the levels of metals in this study with those of other cities of the world (Table 5), this may seem complicated by the disparity in sampling protocols and sample digestion procedure. It can be found that the levels for the metals in dust of our studied centre are lower than other cities, even lower than cities like Tehran and Hong Kong. The only significant exception is Pb, the concentration of which is similar to if not higher than that in those cities used for comparison. This could be attributed to vehicular emission which is common in the city.

**Figure 2:**
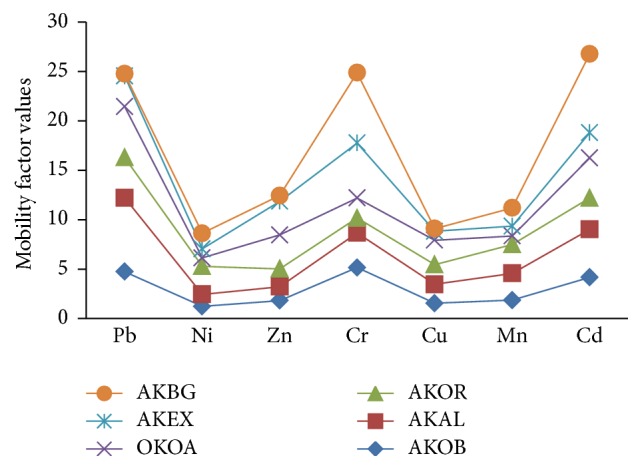
Plot of mobility factors of the studied metals in different sites of Akure.

**Figure 3:**
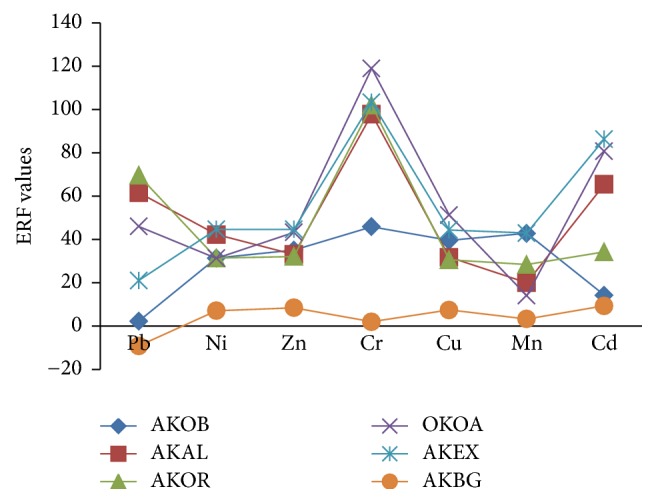
Plot of environmental risk factor values for metals in Akure.

Figures 2 and 3 present the results of bioavailability of the metals in form of mobility and environmental risk factors. It could be observed that the metal mobility followed the order of Pb > Cr > Cd > Mn > Cu > Ni in dust sampled in all sites. The high MF values of these metals are an indication of high biological availability of the metal in dust. Surprisingly, in terms of environmental risk factor (ERF), the highest value was obtained for Cr metal at the sampling sites and closely followed by Cd and Pb. The ERF values for these metals had positive values (greater than 0) in most sites, except at AKBG sampling site where negative values were obtained. This site is the background site where pollution is minimal.

### 3.1. Human Health Risk Assessment

One of the objectives of this work is to ascertain the main route of exposure, which results in health risk to the population dwelling in the area through doses received. To achieve the above objective, the average daily exposure dose (*D*) of the metals and carcinogenic (cancer risk index HI) and noncancer risks (HQs) were calculated to be present in airborne particles, as determined in the atmosphere of Akure sampled sites. To avoid overestimation of the actual health risks, the three most abundant metals in the soluble fractions (Pb, Cr, and Cd) in this study were considered. The adults and children scenarios were assessed to ascertain the human health risks and the results are presented in Table 6.

As it can be seen in the results, ingestion has the highest values of daily exposure doses (*D*) for both adults and children in all the sites of this study. This was closely followed by dermal contact and then inhalation. This is not surprising as airborne particles are less cohesive and more easily resuspended on food and other objects. The observed trend is consistent with previous studies in Nigeria and other urban centres of the world [29, 30]. For Pb, the highest dose of 5.668*E* − 04 of children and adults, respectively, is at AKAL sampling site, a location where government offices are located. This is an indication that Pb pollution in the area is traffic driven. Meanwhile, the highest values of 1.068*E* − 04 and 1.308*E* − 05 doses for children and adults, respectively, were observed for Cr at site AKOA, a residential area, and several welding workshops are located in the area. The observation further supports earlier reports that Cr exhibits very limited ability to penetrate skin and no study of absorption of Cr from soil has been identified. Dermal adsorption of Cd from weathering soil may be even lower and oral absorption of Cd in human is generally reported to be low [31].

The carcinogenic and noncarcinogenic toxicity characterization based on the available metal content showed higher risk for all metals investigated in this study (Table 6). For noncarcinogenic toxicity, the HQ values for Cr metal showed the trend HQ* ingestion* > HQ* inhalation* > HQ* dermal* for both children and adults exposures. The values of HQs for Cr are <1, suggesting an acceptable level of noncarcinogenic adverse health risk in all the sites. However, the HQ* ingestion* values for noncarcinogenic health risk were found to be 1.324*E* − 01, for Pb, at AKEX and AKOB sites, respectively. The highest value was found at AKEX, a high traffic dense area, suggesting that the primary anthropogenic sources of Pb might be traffic emissions. This is an indication that noncarcinogenic adverse health risk of traffic emission in the city should be subject of concern.

The HQ values of metals for children ranged between 0.1622742 and 0.0975528, 0.0414396 and 0.0201285, and 0.0634256 and 0 for Pb, Cr, and Cd, respectively, in studied sites of Akure. However, for adults, the values ranged between 0.002446 and 0.0178378, 0.0028675 and 0.0059035, and zero for Pb, Cr, and Cd, respectively. The highest values obtained for all metals studied in this work for both children and adults were higher than 1.0 × 10^−4^ indicating that human exposure to dust in the city may pose health threat.

## 4. Conclusion

The speciation pattern and toxicity of metals in airborne dust particulate samples in Akure were conducted. The presence of these metals in dust particles poses a significant environmental risk to the people. Experimental results showed the following:

- The sequential extraction revealed that the bulk of the metals in the dust samples collected in this study exists in nonmobile fraction.
- The pollution indices employed revealed that Pb was the most abundant metal, an indication that vehicular emission is the primary source of pollution impacting Akure metropolis.
- Ingestion remains the major route of exposure and metals transfer to human body in our studied sites, although the noncarcinogenic hazard of Pb via inhalation, ingestion, and dermal exposures is lower than the one for children and adults.

## Figures and Tables

**Table 1:** Monitoring locations, their characteristics, and coordinates at Akure.

	Site code	Coordinate	Description	*P*_*i*_ value	Pollution index
(1)	AKEX	N07°17′06.2′′ E005°10′34.0′′	Created along Ilesa-Akure-Owo/Benin road, an expressway, close to the NNPC station	2.0 < *P*_*i*_ < 3.0	Moderate

(2)	AKOA	N07°14′25.3′′ E005°11′15.0′′	Oke-Aro monitoring site, a residential area with many local buildings, close to furniture workshops	*P*_*i*_ > 3.0	Highly contaminated

(3)	AKOB	N07°15′12.7′′ E005°11′43.8′′	Created at the Oja Oba, a market square with heavy traffic	*P*_*i*_ > 3.0	Highly contaminated

(4)	AKAL	N07°14′55.1′′ E005°12′54.4′′	Alagbaka monitoring site, the site of many government offices	2.0 < *P*_*i*_ < 3.0	Considerably contaminated

(5)	AKOR	N07°49′47.2′′ E005°09′37.9′′	Ondo road park monitoring site, a bus terminus with a cluster of petrol dispensing stations	2.0 < *P*_*i*_ < 3.0	Considerably contaminated

(6)	AKBR	N07°13′47.6′′ E005°09′37.9′′	Aponmu monitoring site, a small community and a background site for the study	1.0 < *P*_*i*_ < 2.0	Low contaminated

**Table 2:** Definitions and values of variables used in the estimation of human exposure.

Symbols	Definition	Values [reference]
*C*	Concentrations of metals in dust (mg/kg)	
ABS	Absorption factor (unitless)	0.001 (inorganic) []
AT	Average time (Non carcinogenic ingestion only) yr & day/yr	ED × 365 (noncarcinogenic)
		ED_*n*_ × 365 (noncarcinogenic)
BW	Adult body weight (kg)	70 (adults)
		15 (children)
SL	Skin adherence factor (mgcm^−2^)	0.2 (children)
		0.07 (adults)
CF	Unit conversion factor (kg/mg)	10^−6^
CF_*a*_	Unit conversion factor dermal (kg·cm^2^)/mg·m^2^	0.01
ED	Exposure duration of noncarcinogenic ingestion only (yr)	24 (adults),
		6 (children)
EF	Exposure frequency (day/yr)	350
SA	Adult surface area (cm3)	5700 (adults)
		2800 (children)
IR_ingestion_	Ingesting rate for adults (mg/day)	100 (adults)
		200 (children)
IR_inhalation_	Inhalation rate (m^3^/day)	7.6 (children)
		20 (adults)
PEF	Particulate emission rate (m^3^/kg)	4.28 × 10^9^
RfD_ad_	Absorbed chronic reference dose (mg/kg-day)	Metal specific
RfD_*i*_	Inhalation chronic reference dose (mg/kg-day)	Metal specific
RfD_*o*_	Oral chronic reference dose (mg/kg-day)	Metal specific
SF_ad_	Absorbed dose slope factor (mg/kg-day)^−1^	Metal specific
SF_*i*_	Inhalation slope factor (mg/kg-day)^−1^	Metal specific
SF_*o*_	Oral slope factor (mg/kg-day)^−1^	Metal specific

**Table 3:** Mean values of total metal content (*µ*gg^−1^) ± standard deviation of dust sampled.

Sites	AKEX	AKOA	AKOB	AKAL	AKOR	AKBG
Pb	70.49 ± 16.27	77.11 ± 3.67	65.54 ± 3.13	86.20 ± 5.34	51.82 ± 2.88	11.82 ± 0.88
Cu	3.54 ± 1.05	1.87 ± 3.68	0.62 ± 1.05	0.87 ± 1.05	1.72 ± 1.05	0.54 ± 1.05
Zn	8.23 ± 0.66	11.12 ± 3.17	2.79 ± 3.17	5.13 ± 3.17	9.89 ± 3.17	1.38 ± 3.17
Cr	16.24 ± 1.54	18.57 ± 2.75	10.31 ± 1.54	9.92 ± 1.54	10.01 ± 1.54	9.02 ± 1.54
Ni	2.23 ± 9.12	4.58 ± 1.04	2.63 ± 9.12	5.55 ± 9.12	0.88 ± 9.12	0.87 ± 9.12
Mn	5.81 ± 0.10	1.22 ± 1.07	0.98 ± 0.06	1.48 ± 0.02	1.91 ± 0.05	0.60 ± 0.03
Cd	10.63 ± 1.03	6.06 ± 1.88	6.44 ± 2.01	8.21 ± 0.69	9.53 ± 1.12	3.05 ± 0.09

**Table 4:** The results of analysis of certified and found results for BCR 701 (*µ*gg^−1^).

*µ*gg^−1^	Pb	Cr	Cu	Zn	Ni	Cd	Mn
Step (1)							
Certified	166.9 ± 8.22	87.26 ± 28.4	39.6 ± 3.22	2.6 ± 0.12	6.11 ± 0.22	19.6 ± 2.07	12.5 ± .22
Found	165.2 ± 3.9	103 ± 10.09	32.2 ± 3.9	2.2 ± 0.9	6.09 ± 0.08	19.2 ± 3.00	3.2 ± 3.9

Step (2)							
Certified	108.6 ± 8.22	146.9 ± 22.30	27.83 ± 4.34	31.0 ± 5.0	4.58 ± 0.46	10.09 ± 1.4	8.25 ± 4.3
Found	110.34 ± 9.2	126.6 ± 10.68	29.86 ± 5.53	29.0 ± 7.8	<4.52	9.11 ± 0.90	4.2 ± 8.60

Step (3)							
Certified	97.13 ± 8.10	79.88 ± 5.7	14.18 ± 3.76	119.7 ± 11.2	3.29 ± 0.74	6.90 ± 0.89	4.5 ± 10.2
Found	68.8 ± 9.90	74.29 ± 7.1	<13.11	117.0 ± 10.0	3.56 ± 0.91	6.59 ± 0.70	<3.2

Step (4)							
Certified	102.49 ± 8.2	64.8 ± 8.23	15.46 ± 5.99	56.08 ± 8.01	4.05 ± 0.47	5.84 ± 0.41	7.5 ± 10.3
Found	90.34 ± 9.05	63.09 ± 3.66	15.12 ± 4.52	54.2 ± 7.1	3.59 ± 0.82	4.33 ± 0.60	6.0 ± 13.0

**Table 5:** Comparisons of heavy metal content (*µ*gg^−1^) in street dust in this study with other cities of the world.

Cities	Cd	Cr	Cu	Ni	Pb	Zn	Reference
Birmingham, UK	1.62	-	466.9	41	48	534	[16]
Hong Kong, China	21.8	-	24.8	-	93.4	1.68	[17]
Beijing, China	0.73	80.8	107.7	36.1	71.7	238.6	[18]
Egypt, Egypt,	2.98	58.7	102	38.5	307	1839	[6]
Mubi, Nigeria	0.67	-	25.06	-	207	121	[9]
Akure	2.46	4.83	12.63	6.24	112.45	6.08	This Study

**Table 6:** Health risk from metals in dust for adults and children at various sampled sites of Akure.

			Dinh	Ding	Dder	HQih	HQin	HQder	HI = ∑HQ
AKEX	Pb	Child	1.295*E* − 08	4.635*E* − 04	1.41*E* − 07	3.678*E* − 06	1.324*E* − 01	2.685*E* − 04	0.1326996
		Adult	7.303*E* − 08	4.966*E* − 05	1.981*E* − 07	2.075*E* − 05	0.0141886	3.774*E* − 04	0.0145868
	Cr	Child	2.983*E* − 09	1.068*E* − 04	3.248*E* − 08	1.043*E* − 04	0.0355945	5.413*E* − 04	0.0362402
		Adult	1.683*E* − 08	1.144*E* − 05	4.565*E* − 08	0.0005883	0.0038137	7.608*E* − 04	0.0051628
	Cd	Child	1.952*E* − 09	6.99*E* − 05	2.126*E* − 08	-	0.0698959	0.0008504	-
		Adult	1.101*E* − 08	7.489*E* − 06	2.988*E* − 08	1.101*E* − 06	7.489*E* − 05	0.0011952	0.0012712

AKOA	Pb	Child	1.416*E* − 08	5.07*E* − 04	1.542*E* − 07	4.024*E* − 06	1.44*E* − 01	0.0002938	0.145162
		Adult	7.989*E* − 08	5.432*E* − 05	2.168*E* − 07	2.27*E* − 05	1.552*E* − 01	0.0004129	0.0159567
	Cr	Child	3.411*E* − 09	1.221*E* − 05	3.714*E* − 08	0.0001193	0.0407014	0.000619	0.0414396
		Adult	1.924*E* − 08	1.308*E* − 05	5.22*E* − 08	0.0006727	0.0043609	0.00087	0.0059035
	Cd	Child	1.113*E* − 09	3.985*E* − 05	1.212*E* − 08	1.113*E* − 07	0.0398466	0.0004848	0.0403315
		Adult	6.278*E* − 09	4.269*E* − 06	1.703*E* − 08	-	4.269*E* − 05	3.245*E* − 05	-

AKOB	Pb	Child	1.204*E* − 08	0.0004309	1.311*E* − 07	3.42*E* − 06	0.123128	0.0002497	0.1233811
		Adult	6.79*E* − 08	4.617*E* − 05	1.842*E* − 07	1.929*E* − 05	0.0131923	0.0030705	0.0162821
	Cr	Child	1.894*E* − 09	6.779*E* − 05	2.062*E* − 08	6.621*E* − 05	0.0225973	0.0003437	0.0230071
		Adult	1.068*E* − 08	7.263*E* − 06	2.898*E* − 08	0.0003735	0.0024211	0.0003864	0.003181
	Cd	Child	1.183*E* − 09	4.235*E* − 05	1.288*E* − 08	1.183*E* − 07	0.0423452	0.0005152	0.0428605
		Adult	6.672*E* − 09	4.537*E* − 06	1.81*E* − 08	-	4.537*E* − 05	0.0007241	-

AKAL	Pb	Child	1.583*E* − 08	5.668*E* − 04	1.724*E* − 07	4.498*E* − 06	0.1619413	0.0003284	0.1622742
		Adult	8.931*E* − 08	6.073*E* − 05	2.423*E* − 07	2.537*E* − 05	0.0173509	0.0004615	0.0178378
	Cr	Child	1.822*E* − 09	6.523*E* − 05	1.984*E* − 08	6.371*E* − 05	0.0217425	0.0003307	0.0221368
		Adult	1.028*E* − 08	6.989*E* − 06	2.788*E* − 08	0.0003594	0.0023295	0.0004647	0.0031536
	Cd	Child	1.508*E* − 09	5.398*E* − 05	1.642*E* − 08	1.508*E* − 07	0.0539836	0.0006568	0.0546405
		Adult	8.506*E* − 09	5.784*E* − 06	2.308*E* − 08	-	5.784*E* − 05	0.0009231	-

AKOR	Pb	Child	9.518*E* − 09	0.0003407	1.036*E* − 07	2.704*E* − 06	0.0973526	0.0001974	0.0975528
		Adult	5.369*E* − 08	3.651*E* − 05	1.457*E* − 07	1.525*E* − 05	0.0104306	0.0002775	0.0107233
	Cr	Child	1.839*E* − 09	6.582*E* − 05	2.002*E* − 08	6.429*E* − 05	0.0219397	0.0003337	0.0223377
		Adult	1.037*E* − 08	7.052*E* − 06	2.814*E* − 08	0.0003626	0.0023507	0.000469	0.0031823
	Cd	Child	1.75*E* − 09	6.266*E* − 05	1.906*E* − 08	1.75*E* − 07	0.062663	0.0007624	0.0634256
		Adult	9.873*E* − 09	6.714*E* − 06	2.679*E* − 08	-	6.714*E* − 05	0.0010715	-

AKBR	Pb	Child	2.171*E* − 09	7.772*E* − 05	2.364*E* − 08	6.168*E* − 07	0.0222059	4.503*E* − 05	0.0222515
		Adult	1.225*E* − 08	8.327*E* − 06	3.323*E* − 08	3.479*E* − 06	0.0023792	6.329*E* − 05	0.002446
	Cr	Child	1.657*E* − 09	5.931*E* − 05	1.804*E* − 08	5.793*E* − 05	0.0197699	0.0003007	0.0201285
		Adult	9.345*E* − 09	6.355*E* − 06	2.535*E* − 08	0.0003267	0.0021182	0.0004226	0.0028675
	Cd	Child	5.602*E* − 10	2.005*E* − 05	6.1*E* − 09	5.602*E* − 08	0.0200548	0.000244	0.0202989
		Adult	3.16*E* − 09	2.149*E* − 06	8.573*E* − 09	-	2.149*E* − 05	0.0003429	-
